# Effects of soy and isoflavone interventions on plasma lipid profiles in postmenopausal women: an umbrella review

**DOI:** 10.3389/fnut.2026.1949200

**Published:** 2026-09-22

**Authors:** Jia-He Guo, Zi-Jian Lin, Yue Li, Shuang Zhang, Ming-Li Sun, Lu-Jie Fan, Aykede Abdurazzak, Lian-Kai Wang, Yuan Gao, Ya-Juan Kong, Jia-Ning Jiang, Zeng Wang, Ling-Ling Wang, Yi-Ning Li

**Affiliations:** 1 Department of Ophthalmology, Shengjing Hospital of China Medical University, Shenyang, Liaoning, China; 2 Department of Cardiology, Quanzhou First Hospital Affiliated to Fujian Medical University, Quanzhou, China; 3 Department of Obstetrics and Gynecology, Shengjing Hospital of China Medical University, Shenyang, China; 4 Department of Neurology, Yili Kazakh Autonomous Prefecture Tacheng District People's Hospital, Tacheng, China; 5 Department of Clinical Pharmacology, School of Pharmacy, China Medical University, Shenyang, China; 6 Department of The First Affiliated Hospital, China Medical University, Shenyang, China; 7 School of Public Health, China Medical University, Shenyang, China; 8 Department of Gastroenterology, Shengjing Hospital of China Medical University, Shenyang, China; 9 Center of Reproductive Medicine, Shengjing Hospital of China Medical University, Shenyang, China

**Keywords:** lipid profile, postmenopausal women, randomized controlled trial, soy isoflavones, umbrella review

## Abstract

**Background:**

Although numerous meta-analyses have investigated the effects of soy and isoflavones on lipid profiles in postmenopausal women, a comprehensive evaluation of this evidence remains lacking. This umbrella review synthesized existing meta-analyses of randomized controlled trials to assess the certainty of the evidence.

**Methods:**

We searched PubMed, Web of Science, Embase, and the Cochrane Database for studies published through April 22, 2026. Methodological quality was evaluated using AMSTAR-2, and evidence certainty was assessed using the GRADE approach.

**Results:**

Seven meta-analyses comprising 21 associations were included. According to AMSTAR-2, two meta-analyses (29%) demonstrated moderate or high quality. Eighteen associations were supported by evidence of moderate or high certainty according to GRADE. Soy protein containing isoflavones and soy isoflavone extracts significantly reduced total cholesterol (MD = −0.12 mmol/L, 95% CI: −0.21 to −0.03). Soy isoflavones reduced low-density lipoprotein cholesterol (SMD = −0.11, 95% CI: −0.21 to −0.01). Furthermore, unfermented soy products significantly increased high-density lipoprotein cholesterol (MD = 0.06 mmol/L, 95% CI: 0.02 to 0.10).

**Conclusion:**

Specific soy-based interventions, rather than general soy consumption, were associated with beneficial changes in lipid profiles among postmenopausal women.

**Systematic review registration:**

CRD420251243075.

## 1 Introduction

Soybeans are an excellent food source defined by a complementary nutritional profile, including dietary fiber, high-quality protein, vitamins, unsaturated fatty acids, and a suite of beneficial phytochemicals with antioxidant properties (1). Isoflavones, a class of biologically active phytochemicals abundant in soybeans and their derived products, have garnered significant scientific interest over recent decades (2). These polyphenolic compounds, primarily genistein, daidzein, and glycitein, are structurally analogous to mammalian estrogens, allowing them to function as selective estrogen receptor modulators (SERMs) (3). This unique property underpins their investigation as natural agents for mitigating health conditions linked to declining endogenous estrogen levels, particularly in postmenopausal women (4). Beyond their phytoestrogen activity, isoflavones exhibit potent antioxidant and anti-inflammatory properties, contributing to a broad spectrum of purported physiological benefits (1, 5). Epidemiological observations of lower cardiovascular disease incidence in populations with high soy consumption have fueled extensive research into their cardioprotective potential (6–8). The modulation of the plasma lipid profile, a key modifiable risk factor for atherosclerosis (9), has emerged as a central mechanism through which soy isoflavones may confer cardiovascular benefits.

The specific effect of soy isoflavone intake on the lipid profiles of postmenopausal women has been the subject of substantial randomized controlled trials (RCTs) and subsequent systematic reviews (10). Additionally, major health authorities have recognized the cholesterol-lowering effect of soy protein, which is intrinsically linked to its isoflavone content (11). However, current understandings are primarily grounded in observational evidence synthesized from various study designs, leading to inconsistent conclusions about the health benefits or risks of soy and isoflavones across different outcomes (4, 12). These observed associations are prone to initial overestimation due to methodological biases (13). To mitigate these limitations and arrive at a more balanced assessment, harmonizing evidence from both rigorous RCTs and well-conducted observational studies is essential (14).

Despite lots of primary research and systematic reviews about the effect of soy intake on blood lipids (15–17), the evidence landscape remains fragmented and characterized by inconsistencies. An umbrella review, a systematic synthesis of existing systematic reviews and meta-analyses, is the appropriate methodological response to this challenge (18, 19). It allows for the highest level of evidence integration by mapping the breadth of evidence, explaining discordance between reviews, and critically appraising the methodological rigor of the constituent syntheses (18). Therefore, this umbrella review aims to systematically evaluate and synthesize the evidence from all available systematic reviews and meta-analyses of RCTs on the effect of soy and isoflavone consumption on the plasma lipid profile of postmenopausal women.

## 2 Method

### 2.1 Literature search

To identify relevant systematic reviews with meta-analyses investigating the association between Soy and isoflavone consumption and plasma lipid profile in postmenopausal women, we searched PubMed, Embase, Web of Science, and the Cochrane Database of Systematic Reviews from inception until April 22, 2026. The search strategy is provided in full in the Supplementary Table 1.

Two authors (JH-G and ZJ-L) independently screened records by title/abstract and then by full text without language restrictions. A third author (YN-L) was consulted to resolve any inconsistencies in study selection. To minimize the risk of omission, we supplemented the electronic searches by manually reviewing the bibliographies of all retrieved reviews.

### 2.2 Eligibility criteria

Eligibility for inclusion was defined according to the PICOS framework (Population, Intervention, Comparison, Outcome, Study design) and as following:

(1) Population: Postmenopausal or perimenopausal women;
(2) Intervention: The intervention in this study focuses on soy and isoflavone consumption, which includes several distinct categories. (a) Soy foods referred to conventional soy-derived food products, including unfermented soy products such as tofu, soy milk, and edamame; (b) Soy protein isolate (SPI) referred to isolated soy protein from which isoflavones had been removed or substantially reduced, generally containing at least 90% protein by dry weight; (c) Soy isoflavone supplements referred to preparations containing isolated soy isoflavones, typically administered in tablet or capsule form without accompanying soy protein; and (d) Soy protein containing isoflavones (SPCI) referred to formulations providing both soy protein and naturally retained or added soy isoflavones.
(3) Comparison: This study included two comparison groups, participants who did not consume soybeans or isoflavones, and participants with varying levels of soybean or isoflavone intake;
(4) Outcomes: plasma lipid health outcome; and
(5) Study design: systematic review with meta-analyses of randomized controlled trial.

Exclusion criteria consisted of:

(1) Not a meta-analysis or systematic review with quantitative synthesis;
(2) Not a study focusing on postmenopausal women;
(3) Not a study using blood lipid as the outcome measure;
(4) Interventions did not involve soy, or soy-related subgroup analyses were not performed.

### 2.3 Assessment of study overlap

To assess the overlap of individual RCTs across the included meta-analyses, the Corrected Covered Area (CCA) method was employed. The CCA was calculated using the formula: CCA = (N – r) / (r × c – r) × 100%, where N represents the total number of included primary study instances across all meta-analyses (counting duplicates), r is the number of unique primary studies, and c is the number of meta-analyses. Overlap is categorized as very high (CCA >15%), high (CCA 11-15%), moderate (CCA 6-10%), or slight (CCA 0-5%). If the degree of overlap was high or very high, the systematic review/meta-analysis with the highest quality according to the AMSTAR 2 tool was selected (see below for the description of this tool). If they had the same quality, the most recent one was included (20, 21).

### 2.4 Data extraction

Two reviewers (JH-G and ZJ-L) independently extracted data from eligible studies. All disagreements were resolved by consulting a third reviewer (YN-L). The following data were extracted from each systematic review and meta-analysis: (a) First author's last name, and year of publication; (b) Number of the original studies; (c) Number of participants; (d) Main results; and (e) Methodological quality. Where available, information on the contributing primary RCTs, including the first author and publication year, was extracted for the assessment of study overlap. Study-specific effect estimates and participant numbers were also recorded when reported.

### 2.5 Data analysis

Pooled effect estimates and their corresponding 95% CIs were extracted directly from the eligible meta-analyses. No additional quantitative pooling of effect estimates across meta-analyses was performed. The effect measures and statistical models used in the source meta-analyses were retained, and the results of each meta-analysis were presented separately.

### 2.6 Evaluation of the quality

The methodological quality of each included systematic review and meta-analysis was independently assessed by two reviewers (JH-G and ZJ-L) using the AMSTAR 2 tool. Any disagreements were resolved through discussion and, when necessary, adjudication by a senior reviewer (YN-L). Of the 16 AMSTAR-2 domains, seven were considered critical: protocol registration before commencement of the review, adequacy of the literature search, justification for excluding individual studies, assessment of the risk of bias in individual studies, appropriateness of the meta-analytical methods, consideration of risk of bias when interpreting the findings, and assessment of the presence and likely impact of publication bias. The quality rating according to the AMSTAR 2 tool is as follows: (1) High quality: no or one non-critical weakness. (2) Moderate quality: more than one non-critical weakness. (3) Low quality: one critical flaw with or without non-critical weaknesses. (4) Critically low: More than one critical flaw with or without non critical weaknesses (22).

### 2.7 Assessment of the evidence certainty

The certainty of evidence for each outcome was appraised using the Grading of Recommendations, Assessment, Development and Evaluation (GRADE) framework, which classifies evidence quality into four levels: high, moderate, low, or very low (23, 24). Evidence derived from meta-analyses of RCTs was initially rated as high certainty. The GRADE framework evaluates evidence across five domains that may warrant downgrading (risk of bias, inconsistency, indirectness, imprecision, and publication bias) (25, 26) and three domains that may justify upgrading (large effect, dose-response gradient, and plausible confounding). Risk-of-bias judgments were based on the assessments reported in the source meta-analyses. Evidence was downgraded by one level when more than half of the contributing RCTs were judged to be at high risk of bias. When the source meta-analysis did not report a formal risk-of-bias assessment of the contributing RCTs, the risk-of-bias domain was recorded as unclear; however, the evidence was not downgraded solely because of incomplete reporting. Evidence was downgraded by one level for indirectness when the eligible population included both perimenopausal and postmenopausal women rather than postmenopausal women exclusively. Inconsistency was assessed using the reported *I*^2^ statistic, with 50% < *I*^2^ ≤ 75% and *I*^2^ > 75% considered substantial and considerable heterogeneity, respectively (27). Publication bias was considered present when Egger's or Begg's test was statistically significant (*P* < 0.05) or funnel-plot asymmetry was reported.

## 3 Results

### 3.1 Characteristics of meta-analyses

The database searches identified 2,581 records. After removal of 787 duplicates, 1,794 records underwent title and abstract screening, of which 24 reports were assessed in full text (Figure 1). Seventeen reports were excluded after full-text assessment, with the studies and reasons for exclusion listed in Supplementary Table 2. Seven meta-analyses were included in the final umbrella review (Table 1) (28–34).

**Figure 1:**
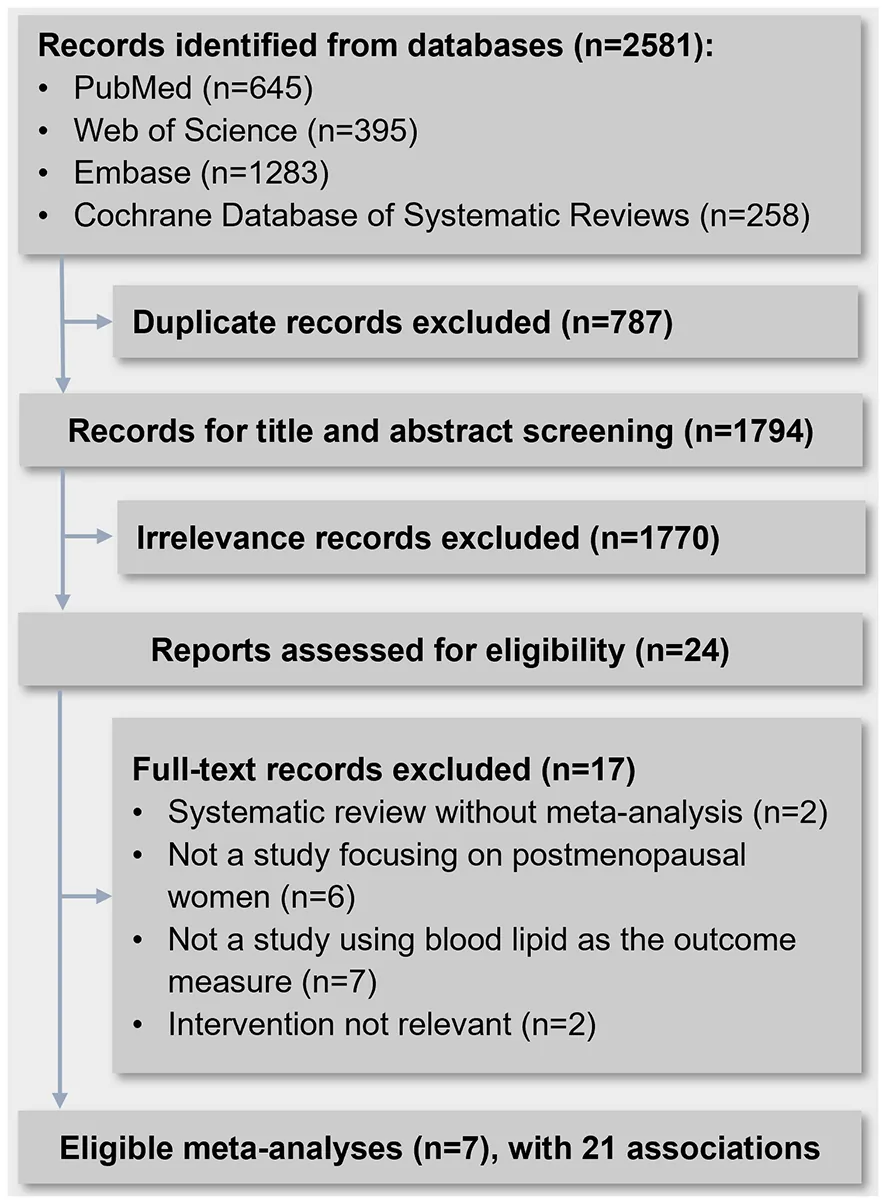
Flow diagram of the study selection process.

**Table 1:** Characteristics of the included meta-analyses evaluating soy-based interventions and plasma lipid profiles in postmenopausal women^a^.

Reference	No. of studies^b^	Sample size^b^	Intervention	Intervention dose	Intervention period (week)	Comparison	Outcome
Yeung et al. (32)	17	853	Mixed (isoflavones, isolated soy protein)	NA	2–26	Placebo	TC
Zhan et al. (33)	23	1,381	Soy protein containing isoflavones	Isoflavone concentrations averaged 80 mg/d	3–26	Control	TC
Taku et al. (31)	19	349	Extracted soy isoflavones	Average: 70 mg/day Range: 27–132 mg/day	4–13	Placebo	LDL-C
Moradi et al. (34)	52	3,456	Soy product	Soy protein: 2–45 g/day	6–96	Placebo	TG, TC, LDL-C, HDL-C, Apolipoprotein A-1, Apolipoprotein B
Bara'nska, et al. (28)	29	2,305	Soy isoflavone extracts and soy protein containing isoflavones	Isoflavones: overall average 87.6 mg/d (median: 80 mg; range: 30.3 to 165 mg)	12	Placebo	TC, TAG, LDL-C, HDL-C
Qi et al. (30)	29	2,457	Unfermented soy product consumption	NA	4–96	Diets without unfermented soy products	TC, TG, LDL-C, HDL-C
Park et al. (29)	61	6,s127	Soy isoflavone	Soy isoflavone: range (40–165 mg/d)	12–144	Placebo	TC, TG, LDL-C

HDL-C, HDL-cholesterol; LDL-C, LDL-cholesterol; TAG, Triacylglycerol; TC, Total cholesterol; TG, Triglycerides. ^a^Eligible soy-specific/postmenopausal subgroup extracted in this umbrella review; ^b^Numbers of studies and participants refer to the overall source meta-analysis.

### 3.2 Overlapping evaluation

The overlap of primary RCTs was assessed between two meta-analyses with partially overlapping intervention scopes. Taku et al. (31) evaluated extracted soy isoflavones, whereas Barańska et al. (28) included both soy isoflavone extracts and soy protein containing isoflavones. To determine whether any primary trials were duplicated across these reviews, a citation matrix was constructed using all identifiable RCTs included in the two meta-analyses (28, 31). The citation matrix comprised 36 unique RCT entries across the two meta-analyses (*N* = 36, *r* = 36, and *c* = 2). No overlapping RCTs were identified, resulting in a CCA of 0%, indicating no overlap. One additional meta-analysis evaluated the same intervention in the same population but reported only a pooled subgroup estimate without a corresponding forest plot or an identifiable list of the contributing RCTs (29). Consequently, it could not be included in the citation matrix or CCA calculation (Supplementary Table 3).

### 3.3 Methodological quality of included meta-analyses

The methodological quality of the seven included meta-analyses was assessed using AMSTAR-2. Of these, one meta-analysis (14%) was rated as high quality, one (14%) as moderate quality, two (28%) as low quality, and three (44%) as critically low quality (Supplementary Table 4).

### 3.4 High certainty of evidence

Six associations were supported by high-certainty evidence, with the findings varying across intervention types and lipid outcomes. For interventions combining isoflavones with isolated soy protein, Yeung et al. (32) found no significant effect on TC (*WMD* = −0.05 mmol/L, 95% *CI*: −0.20 to 0.10). For soy protein containing isoflavones, Zhan et al. (33) reported two separate TC estimates. One indicated a reduction at the threshold of statistical significance (*WMD* = −0.13 mmol/L, 95% *CI*: −0.25 to 0.00), whereas the other showed no significant effect (*WMD* = −0.12 mmol/L, 95% *CI*: −0.29 to 0.05). For interventions comprising soy isoflavone extracts and soy protein containing isoflavones, Barańska et al. (28) found no significant reduction in LDL-C (*MD* = −0.05 mmol/L, 95% *CI*: −0.11 to 0.01). The same meta-analysis also reported a borderline reduction in TAG (*MD* = −0.07 mmol/L, 95% *CI*: −0.14 to 0.00) and a borderline increase in HDL-C (*MD* = 0.03 mmol/L, 95% *CI*: 0.00 to 0.05) (Table 2).

**Table 2:** Summary of associations between soy-based interventions and plasma lipid profiles in postmenopausal women.

References	Intervention	Control	Outcomes	No. of studies	Effect metric	Summary effects (95% CI)	*I^2^* (%)	GRADE
Yeung, et al, (32)	Mixed (isoflavones, isolated soy protein)	Placebo	TC	13	RD	−0.05 (−0.20, 0.10) mmol/L	–	High
Zhan, et al, (33)^a^	Soy protein containing isoflavones	Control	TC	9	WMD	−0.13 (−0.25, 0.00) mmol/L	–	High
Zhan, et al, (33)^b^	Soy protein containing isoflavones	Control	TC	6	WMD	−0.12 (−0.29, 0.05) mmol/L	–	High
Taku, et al, (31)^c^	Soy isoflavones	Placebo	LDL-C	9	WMD	0.03 (−0.11, 0.16) mmol/L	22	Moderate
Moradi, et al, (34)	Soy product	Placebo	TG	49	WMD	−0.06 (−0.11, 0.00) mmol/L	91	Moderate
	Soy product	Placebo	TC	41	WMD	**−0.08 (−0.14**, **−0.01) mmol/L**	92	Moderate
	Soy product	Placebo	LDL-C	49	WMD	**−0.08 (−0.16**, **−0.01) mmol/L**	90	Moderate
	Soy product	Placebo	HDL-C	21	WMD	**−0.06 (−0.11**, **−0.01) mmol/L**	95	Moderate
	Soy product	Placebo	Apo A-1	5	WMD	0.00 (−0.03, 0.04) g/L	59	Low
	Soy product	Placebo	Apo B	5	WMD	−0.06 (−0.15, 0.03) g/L	94	Low
Bara'nska, et al, (28)	Soy isoflavone extracts and soy protein containing isoflavones	Control	TC	28	MD	**−0.12 (−0.21**, **−0.03) mmol/L**	33	Moderate
	Soy isoflavone extracts and soy protein containing isoflavones	Control	LDL-C	27	MD	−0.05 (−0.11, 0.01) mmol/L	5	High
	Soy isoflavone extracts and soy protein containing isoflavones	Control	TAG	28	MD	−0.07 (−0.14, 0.00) mmol/L	49	High
	Soy isoflavone extracts and soy protein containing isoflavones	Control	HDL-C	29	MD	0.03 (0.00, 0.05) mmol/L	19	High
Qi, et al, (30)	Unfermented soy product consumption	Control	TC	29	MD	**−0.25 (−0.39**, **−0.10) mmol/L**	87	Moderate
	Unfermented soy product consumption	Control	LDL–C	29	MD	−0.12 (−0.28, 0.05) mmol/L	92	Moderate
	Unfermented soy product consumption	Control	HDL-C	27	MD	**0.06 (0.02, 0.10) mmol/L**	97	Moderate
	Unfermented soy product consumption	Control	TG	27	MD	**−0.12 (−0.22**, **−0.02) mmol/L**	88	Moderate
Park, et al (29)^c^	Soy isoflavone	Placebo	TC	36	SMD	**−0.10 (−0.19**, **−0.01)**	45	Moderate
	Soy isoflavone	Placebo	TG	35	SMD	**−0.17 (−0.32**, **−0.01)**	67	Very low
	Soy isoflavone	Placebo	LDL-C	35	SMD	**−0.11 (−0.21**, **−0.01)**	49	Moderate

HDL-C, high-density lipoprotein cholesterol; LDL-C, low-density lipoprotein cholesterol; MD, mean difference; RD, risk difference; SMD, standardized mean difference; TAG, triacylglycerol; TC, total cholesterol; TG, triglycerides. ^a^Only postmenopausal; ^b^Postmenopausal with high lipid; ^c^Population includes postmenopausal and perimenopausal women. Bold values indicate statistically significant effect estimates for which the 95% confidence interval does not include the null value of 0.

### 3.5 Moderate certainty of evidence

Twelve associations were supported by moderate-certainty evidence, with findings varying across intervention types and lipid outcomes. For the broad range of soy product interventions evaluated by Moradi et al. (34), a borderline reduction in TG was observed (*WMD* = −0.06 mmol/L, 95% *CI*: −0.11 to 0.00), together with significant reductions in TC (*WMD* = −0.08 mmol/L, 95% *CI*: −0.14 to −0.01) and LDL-C (*WMD* = −0.08 mmol/L, 95% *CI*: −0.16 to −0.01). However, an unfavorable reduction in HDL-C was also reported (*WMD* = −0.06 mmol/L, 95% *CI*: −0.11 to −0.01). For interventions comprising soy isoflavone extracts and soy protein containing isoflavones, Barańska et al. (28) found a significant reduction in TC (*MD* = −0.12 mmol/L, 95% *CI*: −0.21 to −0.03). For unfermented soy product consumption, Qi et al. (30) reported significant reductions in TC (*MD* = −0.25 mmol/L, 95% *CI*: −0.39 to −0.10) and TG (*MD* = −0.12 mmol/L, 95% *CI*: −0.22 to −0.02), as well as a significant increase in HDL-C (*MD* = 0.06 mmol/L, 95% *CI*: 0.02 to 0.10); however, the effect on LDL-C was not significant (*MD* = −0.12 mmol/L, 95% *CI*: −0.28 to 0.05). For soy isoflavone interventions, Taku et al. (31) found no significant effect on LDL-C (*WMD* = 0.03 mmol/L, 95% *CI*: −0.11 to 0.16), whereas Park et al. (29) reported significant reductions in both TC (*SMD* = −0.10, 95% *CI*: −0.19 to −0.01) and LDL-C (*SMD* = −0.11, 95% *CI*: −0.21 to −0.01) (Table 2).

### 3.6 Low and very low certainty of evidence

Two associations were supported by low-certainty evidence, and one was supported by very-low-certainty evidence. For soy product interventions, Moradi et al. (34) reported no significant effects on either apolipoprotein A-I (Apo A-I; *WMD* = 0.00 g/L, 95% *CI*: −0.03 to 0.04) or apolipoprotein B (Apo B; *WMD* = −0.06 g/L, 95% *CI*: −0.15 to 0.03); both associations were rated as low certainty. For soy isoflavone interventions, Park et al. (29) reported a significant reduction in TG (*SMD* = −0.17, 95% *CI*: −0.32 to −0.01), although this association was supported by very-low-certainty evidence and should therefore be interpreted cautiously (Table 2).

### 3.7 Heterogeneity of the included meta-analyses

Among the 21 associations evaluated, 10 (48%) showed statistically significant effects on lipid outcomes. Regarding between-study heterogeneity, 7 associations had low heterogeneity (*I*^2^ < 50%), 2 had moderate heterogeneity (*I*^2^= 50%−75%), and 9 had high heterogeneity (*I*^2^> 75%). Heterogeneity statistics were not reported for the remaining 3 associations (Table 2).

### 3.8 Certainty of evidence

Among the 21 associations evaluated across the included meta-analyses, 6 (28.6%) were supported by high-certainty evidence. These comprised TC for interventions involving isoflavones and isolated soy protein reported by Yeung et al. (32); two TC associations for soy protein containing isoflavones reported by Zhan et al. (33); and LDL-C, TAG, and HDL-C for interventions comprising soy isoflavone extracts and soy protein containing isoflavones reported by Barańska et al. (28). Twelve associations (57.1%) were supported by moderate-certainty evidence. These included TG, TC, LDL-C, and HDL-C for soy product interventions reported by Moradi et al. (34); TC for interventions comprising soy isoflavone extracts and soy protein containing isoflavones reported by Barańska et al. (28); TC, LDL-C, HDL-C, and TG for unfermented soy product consumption reported by Qi et al. (30); LDL-C for soy isoflavone interventions reported by Taku et al. (31); and TC and LDL-C for soy isoflavone interventions reported by Park et al. (29). Evidence was downgraded to moderate certainty primarily because of serious inconsistency, indirectness arising from the inclusion of both perimenopausal and postmenopausal women, or publication bias. Two associations (9.5%), concerning Apo A-I and Apo B following soy product interventions reported by Moradi et al. (34), were supported by low-certainty evidence because of serious inconsistency and imprecision. The remaining association (4.8%), concerning TG following soy isoflavone interventions reported by Park et al. (29), was supported by very-low-certainty evidence because of indirectness, serious inconsistency, and publication bias. Detailed GRADE assessments for all outcomes are presented in Supplementary Table 5.

## 4 Discussion

This umbrella review systematically synthesized and evaluated the evidence regarding the association between soy and isoflavone consumption and plasma lipid profiles in postmenopausal women. According to the GRADE criteria, six associations were graded as high certainty evidence. Specifically, high-quality evidence supported the following associations: the effect of isoflavones and isolated soy protein on TC, soy protein containing isoflavones on TC, soy isoflavone extracts and soy protein containing isoflavones on LDL-C, TAG, and HDL-C.

The effects of soy-based interventions on TC varied according to intervention type. Significant reductions were reported by Moradi et al. for a broad range of soy-based interventions, by Barańska et al. for interventions comprising soy isoflavone extracts and soy protein containing isoflavones, by Qi et al. for interventions classified as unfermented soy product consumption (30), and by Park et al. for soy isoflavone interventions (29). By contrast, Yeung et al. found no significant effect of interventions involving isoflavones and isolated soy protein (32). Zhan et al. reported two estimates for soy protein containing isoflavones: one was at the threshold of statistical significance, whereas the other was not statistically significant (33). These differences may reflect variation in intervention composition, baseline lipid status, isoflavone and soy protein doses, intervention duration, and comparator conditions. Subgroup analyses by Qi et al. suggested that TC responses differed according to participants' baseline lipid status, although the considerable heterogeneity in their overall analysis warrants cautious interpretation. Mechanistically, soy isoflavones may influence lipid metabolism through their interactions with estrogen receptors, which could be particularly relevant after the decline in endogenous estrogen accompanying menopause (35). When provided with soy protein, isoflavones may also act alongside soy-derived peptides and other constituents that regulate hepatic cholesterol synthesis and clearance (36). However, mechanisms involving phospholipids, phytosterols, dietary fiber, and the displacement of foods rich in saturated fat are more applicable to whole-soy-food interventions than to isolated isoflavone supplements. These mechanisms therefore cannot be assumed to apply equally across all intervention categories. Overall, the observed heterogeneity suggests that the TC-lowering effect depends on both the composition of the soy intervention and the characteristics of the study population.

The effects of soy-based interventions on LDL-C varied according to the intervention type. Moradi et al. (34) reported a small but statistically significant reduction in LDL-C following mixed soy-based intervention, although considerable heterogeneity was observed. Park et al. (29) likewise reported a small reduction following soy isoflavone supplementation. By contrast, Taku et al. (31) found no significant effect of extracted soy isoflavones on LDL-C, while Barańska et al. (28) reported a small but non-significant reduction following interventions comprising soy isoflavone extracts and soy protein containing isoflavones. Similarly, Qi et al. (30) found no significant overall effect of unfermented soy product consumption on LDL-C, accompanied by considerable heterogeneity. However, subgroup analyses by Qi et al. suggested greater LDL-C reductions among women older than 56 years, those with lipid disorders, and those receiving soy protein containing isoflavones rather than isolated soy protein or isolated soy isoflavones. These subgroup findings suggest that baseline lipid status, age, and intervention composition may modify the LDL-C response, although they should be interpreted cautiously because of their exploratory nature. Mechanistically, the soy protein component may contribute to LDL-C reduction through bioactive peptides and the 7S globulin fraction, which have been proposed to enhance hepatic LDL-receptor expression and regulate cholesterol homeostasis (37). Isoflavones may additionally modulate lipid metabolism through estrogen receptor-dependent pathways (38). Thus, the concurrent provision of soy protein and isoflavones may produce additive effects that are not consistently observed with isolated isoflavone supplementation. Differences in isoflavone composition and bioavailability, intervention dose and duration, comparator diets, and participant characteristics may further explain the inconsistent findings across meta-analyses.

The effects of soy-based interventions on HDL-C appeared to vary according to intervention composition. Qi et al. (30) reported a significant increase in HDL-C following interventions classified as unfermented soy product consumption, whereas Barańska et al. (28) observed a smaller, borderline increase following interventions comprising soy isoflavone extracts and soy protein containing isoflavones. By contrast, Moradi et al. reported a significant reduction in HDL-C in their overall analysis of interventions comprising isolated soy protein, isolated soy isoflavones, soy protein containing isoflavones, and other dietary soy preparations; this association was rated as moderate-certainty evidence in our GRADE assessment (34). These apparently divergent findings may partly reflect differences in the composition and distribution of the interventions included in each meta-analysis. Notably, the category of unfermented soy products used by Qi et al. encompassed several preparations, including isolated soy protein, isolated soy isoflavones, and soy protein containing isoflavones, rather than exclusively conventional whole soy foods. Their subgroup analysis indicated that soy protein containing isoflavones was associated with a greater increase in HDL-C than isolated soy protein or isolated soy isoflavones, suggesting that the combined provision of soy protein and isoflavones may be more relevant than either component alone. In Moradi et al., however, the significant reduction in HDL-C was observed specifically in the isolated soy protein subgroup, whereas the effects of the other intervention categories were not significant. Moradi et al. also found that HDL-C responses differed according to baseline lipid status, isoflavone dose, intervention duration, and whether soy was provided as a supplement or as part of the diet. Therefore, the contrasting pooled estimates should not be interpreted as demonstrating an inherent benefit of one broadly defined soy intervention over another. Differences in intervention classification, participant characteristics, baseline lipid status, dose, duration, and comparator conditions may all have contributed to the inconsistent findings. Furthermore, the considerable heterogeneity reported by Qi et al. and Moradi et al. indicates that these HDL-C findings should be interpreted cautiously.

Although several statistically significant reductions in TC and LDL-C were identified, their absolute magnitudes were generally modest. Accordingly, these lipid changes alone would be expected to confer only a limited short-term reduction in cardiovascular risk, particularly at the individual level. Nevertheless, even small reductions in LDL-C may contribute incrementally to cardiovascular prevention when they are sustained over time and incorporated into an overall cardioprotective dietary pattern. Evidence from large-scale lipid-lowering trials indicates that the proportional reduction in major vascular events is related to the absolute magnitude of LDL-C reduction (39), while genetic and clinical evidence suggests that the cardiovascular benefit also depends on the duration of exposure to lower LDL-C levels (40). Therefore, soy-based dietary interventions should be regarded as a potentially useful component of a comprehensive dietary strategy rather than as a substitute for established lipid-lowering therapies in individuals requiring pharmacological treatment. Moreover, because the available trials predominantly evaluated intermediate lipid outcomes over relatively short intervention periods, it remains uncertain whether these modest improvements translate into meaningful reductions in cardiovascular events. Larger, longer-duration randomized controlled trials assessing clinical cardiovascular outcomes are warranted to clarify their long-term clinical relevance.

Apart from the dietary intake of soybeans which has an impact on blood lipids, other special dietary patterns can also have a certain effect in lowering blood lipids. For instance, a recent meta-analysis of randomized controlled trials on ketogenic diets reported reductions in triglycerides alongside increases in LDL-C and total cholesterol, highlighting a contrasting lipid profile response (41). Similarly, capsaicin- and pepper-based interventions have been shown to reduce total cholesterol and triglycerides, with limited effects on LDL-C and HDL-C (42). In contrast, phytosterol supplementation demonstrates a more pronounced lipid-lowering effect, particularly on LDL-C and total cholesterol, especially at doses more than 2 g/day and in hypercholesterolemic populations (43). Likewise, dietary fiber interventions such as guar gum have been associated with substantial reductions in total cholesterol and LDL-C, particularly among individuals with metabolic disorders (44). Taken together, these comparisons suggest that soy isoflavones exert moderate lipid-lowering effects relative to other dietary interventions, with a more balanced impact across lipid parameters. This profile may be particularly relevant for postmenopausal women, where combined metabolic and hormonal mechanisms are involved. Therefore, while the magnitude of change may be smaller than that observed with targeted interventions such as phytosterols or soluble fibers, the overall cardiometabolic benefit of soy is likely mediated through complementary mechanisms, including phytoestrogenic activity and modulation of cholesterol metabolism.

Several potential limitations of this umbrella review should be considered. First, although the included meta-analyses were based on RCTs, substantial heterogeneity was observed across many associations. This high heterogeneity may be explained by several factors. These include differences in intervention characteristics (e.g., type of soy product, isoflavone dose, and duration of supplementation), variability in participant populations (such as baseline lipid status, menopausal stage, and comorbidities), and methodological diversity across trials. Second, this review relied on previously published systematic reviews and meta-analyses. Although it is possible that some individual RCTs were not captured, the use of a comprehensive search strategy across multiple databases and manual reference screening likely minimized this risk. Third, meta-analyses that did not conduct quantitative synthesis were excluded, which may have led to an incomplete representation of all available evidence. Nevertheless, we summarized findings from a wide range of studies to ensure a comprehensive overview. Despite these limitations, this umbrella review provides a systematic and evidence-graded synthesis of current knowledge, highlighting both consistent beneficial effects of soy and isoflavones on specific lipid parameters and critical gaps requiring further investigation.

## 5 Conclusion

Based on this umbrella review, specific soy-based interventions were associated with modest changes in TC, LDL-C, TG, and HDL-C; however, the direction and magnitude of these associations varied according to intervention composition and study population. Further high-quality meta-analyses and well-designed randomized controlled trials are warranted to confirm these associations.

## Data Availability

The original contributions presented in the study are included in the article/Supplementary material, further inquiries can be directed to the corresponding authors.
