# Supplementary material for: Effects of soy and isoflavone interventions on plasma lipid profiles in postmenopausal women: an umbrella review
Source: Front Nutr. 2026 Sep 22;13:1949200. doi: 10.3389/fnut.2026.1949200 (PMC13638521; doi:10.3389/fnut.2026.1949200)
Supplement: Supplementary file 1 [file Table_1.docx]

**Supplementary Table 1. Search strategy from database inception to April 22, 2026**

| **Database** | **Search term** | **Results** |
| --- | --- | --- |
| PubMed | #1 (((((((randomized controlled trial) OR (controlled clinical trial)) OR (randomized trial)) OR (placebo)) OR (clinical trials)) OR (randomly)) OR (trial)) OR (Randomized Clinical Trial)  #2 ((systematic review) OR (meta-analysis)) OR (meta-analyses)  #3 ((((((((((((((((Soybean Proteins) OR (Soybeans)) OR (soy foods)) OR (Soy Proteins)) OR (Soybean Protein)) OR (Soybean)) OR (soy food)) OR (Soy Protein)) OR (natto)) OR (Tofu)) OR (miso)) OR (Tempeh)) OR (Soy)) OR (Isoflavone)) OR (soya)) OR (Phytoestrogen)) OR (soymilk)  #4 #1 AND #2 AND #3 | 645 |
| Web of Science | #1 (ALL=("randomized controlled trial") OR ALL=("controlled clinical trial") OR ALL=("randomized trial") OR ALL=("placebo") OR ALL=("clinical trials") OR ALL=("randomly") OR ALL=("trial") OR ALL=("Randomized Clinical Trial"))  #2 ((ALL=("systematic review")) OR ALL=("meta-analysis") OR ALL=("meta-analyses"))  #3 ((ALL=("Soybean Proteins")) OR ALL=("Soybeans") OR ALL=("soy foods") OR ALL=("Soy Proteins") OR ALL=("Soybean Protein") OR ALL=("Soybean") OR ALL=("soy food") OR ALL=("Soy Protein") OR ALL=("natto") OR ALL=("Tofu") OR ALL=("Tempeh") OR ALL=("miso") OR ALL=("soy") OR ALL=("isoflavone") OR ALL=("soya") OR ALL=("Phytoestrogen") OR ALL=("soymilk"))  #4 #1 AND #2 AND #3 | 395 |
| Embase | #1 'randomized controlled trial'  #2 'controlled clinical trial'  #3 'randomized trial'  #4 'placebo'  #5 'clinical trials'  #6 'randomly'  #7 'trial'  #8 'randomized clinical trial'  #9 #1 OR #2 OR #3 OR #4 OR #5 OR #6 OR #7 OR #8  #10 'systematic review'  #11 'meta-analysis'  #12 'meta-analyses'  #13 #10 OR #11 OR #12  #14 'soybean proteins'  #15 'soybeans'  #16 'soy foods'  #17 'soy proteins'  #18 'soybean protein'  #19 'soybean'  #20 'soy food'  #21 'soy protein'  #22 'natto'  #23 'tofu'  #24 'miso'  #25 'tempeh'  #26 'soy'  #27 'isoflavone'  #28 'soya'  #29 'phytoestrogen'  #30 'soymilk'  #31 #14 OR #15 OR #16 OR #17 OR #18 OR #19 OR #20 OR #21 OR #22 OR #23 OR #24 OR #25 OR #26 OR #27 OR #28 OR #29 OR #30  #32 #9 AND #13 AND #31 | 1283 |
| Cochrane Database of Systematic Review | #1 (Phytoestrogen) OR (soymilk)  #2 (miso) OR (Tempeh) OR (Soy) OR (Isoflavone) OR (soya)  #3 (Soybean) OR (soy food) OR (Soy Protein) OR (natto) OR (Tofu)  #4 (Soybean Proteins) OR (Soybeans) OR (soy foods) OR (Soy Proteins) OR (Soybean Protein)  #5 #1 OR #2 OR #3 OR #4  #6 (systematic review) OR (meta-analysis) OR (meta-analyses)  #7 (randomly) OR (trial) OR (Randomized Clinical Trial)  #8 (randomized controlled trial) OR (controlled clinical trial) OR (randomized trial) OR (placebo) OR (clinical trials)  #9 #7 OR #8  #10 #5 AND #6 AND #9 | 258 |
| **Total** |  | **2581** |

**Supplementary Table 2. List of studies excluded after full-text assessment and reasons for exclusion**

| **Reasons for exclusion** | **List of studies** |
| --- | --- |
| Systematic review without meta-analysis (n=2) | 1. DiNubile, Nicholas A. “A potential role for avocado- and soybean-based nutritional supplements in the management of osteoarthritis: a review.” *The Physician and sportsmedicine* vol. 38,2 (2010): 71-81. doi:10.3810/psm.2010.06.1785 2. Akhavan Zanjani, Mohsen et al. “Soy Foods and the Risk of Fracture: A Systematic Review of Prospective Cohort Studies.” “Soja-Lebensmittel und Frakturrisiko: eine systematische Übersicht über prospektive Kohortenstudien.” *Complementary medicine research* vol. 29,2 (2022): 172-181. doi:10.1159/000519036 |
| Not a study focusing on postmenopausal women (n=6) | 1. Maleki Sedgi, Fatemeh et al. “Effect of fermented soybean on metabolic outcomes, anthropometric indices, and body composition: a systematic review and meta-analysis of clinical trials.” *Food & function* vol. 16,2 389-405. 20 Jan. 2025, doi:10.1039/d4fo02668c 2. Zare, Reza et al. “Effect of Soy Protein Supplementation on Muscle Adaptations, Metabolic and Antioxidant Status, Hormonal Response, and Exercise Performance of Active Individuals and Athletes: A Systematic Review of Randomised Controlled Trials.” *Sports medicine (Auckland, N.Z.)* vol. 53,12 (2023): 2417-2446. doi:10.1007/s40279-023-01899-w 3. Asbaghi, Omid et al. “The Effects of Soy Products on Cardiovascular Risk Factors in Patients with Type 2 Diabetes: A Systematic Review and Meta-analysis of Clinical Trials.” *Advances in nutrition (Bethesda, Md.)* vol. 13,2 (2022): 455-473. doi:10.1093/advances/nmab121 4. Barańska, Agnieszka et al. “Effects of Soy Isoflavones on Glycemic Control and Lipid Profile in Patients with Type 2 Diabetes: A Systematic Review and Meta-Analysis of Randomized Controlled Trials.” *Nutrients* vol. 13,6 1886. 31 May. 2021, doi:10.3390/nu13061886 5. Wang, Xue et al. “Effects of soy isoflavone supplementation on patients with diabetic nephropathy: a systematic review and meta-analysis of randomized controlled trials.” *Food & function* vol. 12,17 (2021): 7607-7618. doi:10.1039/d1fo01175h 6. Eslami, Omid, and Farzad Shidfar. “Soy milk: A functional beverage with hypocholesterolemic effects? A systematic review of randomized controlled trials.” *Complementary therapies in medicine* vol. 42 (2019): 82-88. doi:10.1016/j.ctim.2018.11.001 |
| Not a study using blood lipid as the outcome measure (n=7) | 1. Taku, Kyoko et al. “Extracted or synthesized soybean isoflavones reduce menopausal hot flash frequency and severity: systematic review and meta-analysis of randomized controlled trials.” *Menopause (New York, N.Y.)* vol. 19,7 (2012): 776-90. doi:10.1097/gme.0b013e3182410159 2. Viscardi, Gabrielle et al. “Effect of Soy Isoflavones on Measures of Estrogenicity: A Systematic Review and Meta-Analysis of Randomized Controlled Trials.” *Advances in nutrition (Bethesda, Md.)* vol. 16,1 (2025): 100327. doi:10.1016/j.advnut.2024.100327 3. Luan, Haodi et al. “Effects of soy isoflavones on menopausal symptoms in perimenopausal women: a systematic review and meta-analysis.” *PeerJ* vol. 13 e19715. 23 Jul. 2025, doi:10.7717/peerj.19715 4. Otun, Jemiliat et al. “Systematic Review and Meta-analysis on the Effect of Soy on Thyroid Function.” *Scientific reports* vol. 9,1 3964. 8 Mar. 2019, doi:10.1038/s41598-019-40647-x 5. Duan, Ying et al. “Soy consumption and serum uric acid levels: A systematic review and meta-analysis.” *Frontiers in nutrition* vol. 9 975718. 2 Sep. 2022, doi:10.3389/fnut.2022.975718 6. Khodarahmi, Mahdieh et al. “A systematic review and meta-analysis of the effects of soy on serum hs-CRP.” *Clinical nutrition (Edinburgh, Scotland)* vol. 38,3 (2019): 996-1011. doi:10.1016/j.clnu.2018.09.007 7. Wang, Chenting et al. “Soy Product Consumption and the Risk of Cancer: A Systematic Review and Meta-Analysis of Observational Studies.” *Nutrients* vol. 16,7 986. 28 Mar. 2024, doi:10.3390/nu16070986 |
| Intervention not relevant (n=2) | 1. Zhou, Shuaishuai et al. “Effects of high-quality protein supplementation on cardiovascular risk factors in individuals with metabolic diseases: A systematic review and meta-analysis of randomized controlled trials.” *Clinical nutrition (Edinburgh, Scotland)* vol. 43,8 (2024): 1740-1750. doi:10.1016/j.clnu.2024.06.013 2. Wolters, Maike et al. “Effects of phytoestrogen supplementation on intermediate cardiovascular disease risk factors among postmenopausal women: a meta-analysis of randomized controlled trials.” *Menopause (New York, N.Y.)* vol. 27,9 (2020): 1081-1092. doi:10.1097/GME.0000000000001566 |

**Supplementary Table 3. Citation matrix of meta-analyses.**

| **Original RCT** | **Taku, 2008, (1)** | **Barańska, 2021, (2)** |
| --- | --- | --- |
| Allen, 2007 |  | √ |
| Aubertin-Leheudre, 2008 |  | √ |
| Barasa, 2018 |  | √ |
| Basaria, 2009 |  | √ |
| Campbell, 2010 |  | √ |
| Cheng, 2007 | √ |  |
| Chilibeck, 2013 |  | √ |
| Choubette, 2011 |  | √ |
| Colacurci, 2005 |  | √ |
| Dalais, 2003 |  | √ |
| Gallagher, 2004 (52 mg) and 2004 (96 mg) |  | √ |
| Gardner, 2001 |  | √ |
| Garrido, 2006 |  | √ |
| Gonzalez, 2007 | √ |  |
| Hall, 2006 | √ |  |
| Han, 2002 |  | √ |
| Ho, 2007 (40 mg) and 2007 (80 mg) |  | √ |
| Jassi, 2010 |  | √ |
| Kim, 2013 |  | √ |
| Kreijkamp-Kaspers, 2004 |  | √ |
| Liu, 2012 |  | √ |
| Mackey, 2000 |  | √ |
| Mangano, 2013 |  | √ |
| Nahas, 2007 |  | √ |
| Nestel, 1997 | √ |  |
| Nikander, 2004 | √ |  |
| Öztürk Turhan, 2009 |  | √ |
| Rios, 2008 |  | √ |
| Sathyapalan, 2018 |  | √ |
| Simons, 2000 | √ |  |
| Teede, 2005 |  | √ |
| Uesugi, 2002 | √ |  |
| Uesugi, 2004 | √ |  |
| Vigna, 2000 |  | √ |
| Wu, 2006 |  | √ |
| Yildiz, 2005 | √ |  |
|  |  |  |
| N | 36 |  |
| Rows (r) | 36 |  |
| Columns (c) | 2 |  |
| Corrected Covered Area= (N-r)/(rc-r) | 0%. No overlap. |  |

**Supplementary Table 4. Methodological quality according to AMSTAR 2 of the included systematic reviews and meta-analyses.**

| Author, year, ref | Q1 | Q2* | Q3 | Q4* | Q5 | Q6 | Q7* | Q8 | Q9* | Q10 | Q11* | Q12 | Q13* | Q14 | Q15* | Q16 | Quality |
| --- | --- | --- | --- | --- | --- | --- | --- | --- | --- | --- | --- | --- | --- | --- | --- | --- | --- |
| Yeung, 2003, (3) | Yes | No | No | Yes | Yes | Yes | Yes | Yes | No | No | Yes | No | No | Yes | Yes | Yes | Critically low |
| Zhan, 2005, (4) | Yes | No | No | No | No | No | Partial Yes | Yes | No | No | Yes | No | No | Yes | Yes | No | Critically low |
| Taku, 2008, (1) | Yes | No | No | Yes | Yes | Yes | Yes | Yes | Yes | No | Yes | Yes | Yes | Yes | Yes | Yes | Low |
| Moradi, 2019, (5) | Yes | No | Yes | Yes | Yes | No | No | Yes | Yes | No | Yes | Yes | No | Yes | Yes | Yes | Critically low |
| Barańska, 2021, (2) | Yes | No | No | Yes | Yes | Yes | Yes | Yes | Yes | No | Yes | No | Yes | Yes | Yes | Yes | Low |
| Qi, 2024, (6) | Yes | Yes | No | Yes | Yes | Yes | Yes | Yes | Yes | No | Yes | No | Yes | Yes | Yes | Yes | Moderate |
| Park, 2025, (7) | Yes | Yes | Yes | Yes | Yes | Yes | Yes | Yes | Yes | No | Yes | Yes | Yes | Yes | Yes | Yes | High |

*****Critical questions; **Q:** question; **Q1:** Research question and inclusion criteria; **Q2:** Protocol prior to the conduct the review; **Q3:** Explanation of the selection of the study designs; **Q4:** Literature search strategy; **Q5:** Study selection in duplicate; **Q6:** Data extraction in duplicate; **Q7:** List of excluded studies and justify the exclusions; **Q8:** Description of the included studies in detail; **Q9:** Assessing the risk of bias in individual studies included; **Q10:** Sources of funding of the studies included; **Q11:** Appropriate methods for statistical combination of results; **Q12**: Assessing of the impact of risk of bias in individual studies included on the results; **Q13:** Interpreting of results taking into account the risk of bias in individual studies included; **Q14:** Discussion of the heterogeneity of results; **Q15:** Evaluation and discussion of the publication bias; **Q16:** Potential sources of conflict of interest of the review. **Cite:** Shea BJ, et al. AMSTAR 2: a critical appraisal tool for systematic reviews that include randomised or non-randomised studies of healthcare interventions, or both. BMJ. 2017 Sep 21;358:j4008.
**Supplementary Table 5. GRADE assessment of soy-specific associations with plasma lipid profiles**

| **Author, year, ref** | **Intervention** | **Outcomes** | **Downgrade factors** | | | | | **Upgrade factors** | | | **GRADE** |
| --- | --- | --- | --- | --- | --- | --- | --- | --- | --- | --- | --- |
|  |  |  | **Risk of bias** ^c^ | **Indirectness** | **Inconsistency** | **Imprecision** | **Publication bias** | **Large effect** | **Dose-response** | **Plausible confounding** |  |
| Yeung, 2003, (3) | Mixed (isoflavones, isolated soy protein) | TC | Not reported ^i^ | No serious | No serious | No serious | No serious | No | No | No | High |
| Zhan, 2005, (4) ^a^ | Soy protein containing isoflavones | TC | Not reported ^i^ | No serious | No serious | No serious | No serious | No | No | No | High |
| Zhan, 2005, (4) ^b^ | Soy protein containing isoflavones | TC | Not reported ^i^ | No serious | No serious | No serious | No serious | No | No | No | High |
| Taku, 2008, (1) | Soy isoflavones | LDL-C | No serious | Serious limitation ^d^ | No serious | No serious | No serious | No | No | No | Moderate |
| Moradi, 2019, (5) | Soy product | TG | No serious | No serious | Serious limitation ^f^ | No serious | No serious | No | No | No | Moderate |
| Moradi, 2019, (5) | Soy product | TC | No serious | No serious | Serious limitation ^f^ | No serious | No serious | No | No | No | Moderate |
| Moradi, 2019, (5) | Soy product | LDL-C | No serious | No serious | Serious limitation ^f^ | No serious | No serious | No | No | No | Moderate |
| Moradi, 2019, (5) | Soy product | HDL-C | No serious | No serious | Serious limitation ^f^ | No serious | No serious | No | No | No | Moderate |
| Moradi, 2019, (5) | Soy product | Apo A-1 | No serious | No serious | Serious limitation ^e^ | Serious limitation ^g^ | No serious | No | No | No | Low |
| Moradi, 2019, (5) | Soy product | Apo B | No serious | No serious | Serious limitation ^f^ | Serious limitation ^g^ | No serious | No | No | No | Low |
| Barańska, 2021, (2) | Soy isoflavone extracts and soy protein containing isoflavones | TC | No serious | No serious | No serious | No serious | Serious limitation ^h^ | No | No | No | Moderate |
| Barańska, 2021, (2) | Soy isoflavone extracts and soy protein containing isoflavones | LDL-C | No serious | No serious | No serious | No serious | No serious | No | No | No | High |
| Barańska, 2021, (2) | Soy isoflavone extracts and soy protein containing isoflavones | TAG | No serious | No serious | No serious | No serious | No serious | No | No | No | High |
| Barańska, 2021, (2) | Soy isoflavone extracts and soy protein containing isoflavones | HDL-C | No serious | No serious | No serious | No serious | No serious | No | No | No | High |
| Qi, 2024, (6) | Unfermented soy product consumption | TC | No serious | No serious | Serious limitation ^f^ | No serious | No serious | No | No | No | Moderate |
| Qi, 2024, (6) | Unfermented soy product consumption | LDL-C | No serious | No serious | Serious limitation ^f^ | No serious | No serious | No | No | No | Moderate |
| Qi, 2024, (6) | Unfermented soy product consumption | HDL-C | No serious | No serious | Serious limitation ^f^ | No serious | No serious | No | No | No | Moderate |
| Qi, 2024, (6) | Unfermented soy product consumption | TG | No serious | No serious | Serious limitation ^f^ | No serious | No serious | No | No | No | Moderate |
| Park, 2025, (7) | Soy isoflavone | TC | No serious | Serious limitation ^d^ | No serious | No serious | No serious | No | No | No | Moderate |
| Park, 2025, (7) | Soy isoflavone | TG | No serious | Serious limitation ^d^ | Serious limitation ^e^ | No serious | Serious limitation ^h^ | No | No | No | Very low |
| Park, 2025, (7) | Soy isoflavone | LDL-C | No serious | Serious limitation ^d^ | No serious | No serious | No serious | No | No | No | Moderate |

^a^ Only postmenopausal;

^b^ Postmenopausal with high lipid;

^c^ Evidence certainty was downgraded by one level when more than half of the individual RCTs included in the source meta-analysis were judged to be at high risk of bias, based on the risk-of-bias assessments reported in the original review;

^d^ Evidence certainty was downgraded by one level for indirectness when the population included both postmenopausal and perimenopausal women rather than postmenopausal women exclusively.

^e^ Substantial heterogeneity was observed across studies (50% < *I^2^* <= 75%); therefore, the certainty of evidence was downgraded by one level;

^f^ Considerable heterogeneity was observed across studies (*I^2^* > 75%); however, the heterogeneity could be explained, and the certainty of evidence was therefore downgraded by one level;

^g^ Total number of participants was insufficient to detect a meaningful effect, the certainty of evidence was downgraded by one level;

^h^ Evidence of publication bias was detected (Egger’s test P < 0.05, Begg’s test P < 0.05, or funnel plot asymmetry); therefore, the certainty of evidence was downgraded by one level.

^i^ Risk of bias was considered unclear when the source meta-analysis did not report a formal assessment of the contributing RCTs; consistent with GRADE guidance, evidence was not downgraded solely because of incomplete reporting.

**References**

1. Taku K, Umegaki K, Ishimi Y, Watanabe S. Effects of extracted soy isoflavones alone on blood total and LDL cholesterol: Meta-analysis of randomized controlled trials. Ther Clin Risk Manag. 2008 Oct;4(5):1097–103. doi:10.2147/tcrm.s3262. Cited in: Pubmed; PMID 19209289.

2. Baranska A, Blaszczuk A, Kanadys W, Baczewska B, Jedrych M, Wawryk-Gawda E, Polz-Dacewicz M. Effects of Soy Protein Containing of Isoflavones and Isoflavones Extract on Plasma Lipid Profile in Postmenopausal Women as a Potential Prevention Factor in Cardiovascular Diseases: Systematic Review and Meta-Analysis of Randomized Controlled Trials. Nutrients. 2021 Jul 24;13(8). doi:10.3390/nu13082531. Cited in: Pubmed; PMID 34444691.

3. Yeung J, Yu TF. Effects of isoflavones (soy phyto-estrogens) on serum lipids: a meta-analysis of randomized controlled trials. Nutr J. 2003 Nov 19;2:15. Epub 20031119. doi:10.1186/1475-2891-2-15. Cited in: Pubmed; PMID 14627440.

4. Zhan S, Ho SC. Meta-analysis of the effects of soy protein containing isoflavones on the lipid profile. Am J Clin Nutr. 2005 Feb;81(2):397–408. doi:10.1093/ajcn.81.2.397. Cited in: Pubmed; PMID 15699227.

5. Moradi M, Daneshzad E, Azadbakht L. The effects of isolated soy protein, isolated soy isoflavones and soy protein containing isoflavones on serum lipids in postmenopausal women: A systematic review and meta-analysis.(1549-7852 (Electronic)). eng.

6. Qi J, Zhu R, Mao J, Wang X, Xu H, Guo L. Effect of Unfermented Soy Product Consumption on Blood Lipids in Postmenopausal Women: A Systematic Review and Meta-Analysis of Randomized Controlled Trials. J Acad Nutr Diet. 2024 Nov;124(11):1474–1491 e1. Epub 20240209. doi:10.1016/j.jand.2024.02.006. Cited in: Pubmed; PMID 38342411.

7. Park SH, Oh MR, Lee J, Choi HK, Hwang JT. Effects of Plant-Derived Dietary Supplements on Lipid Profiles in Menopausal Women: An Updated Systematic Review and Meta-Analysis of Randomized Placebo-Controlled Trials. Phytother Res. 2025 Aug;39(8):3486–3507. Epub 20250704. doi:10.1002/ptr.8511. Cited in: Pubmed; PMID 40613540.
